# Adiponectin Protects Hypoxia/Reoxygenation-Induced Cardiomyocyte Injury by Suppressing Autophagy

**DOI:** 10.1155/2022/8433464

**Published:** 2022-10-17

**Authors:** Jia Guo, Kaiyi Zhu, Zhidong Li, Chuanshi Xiao

**Affiliations:** ^1^Center for Hypertension Care, Shanxi Medical University First Hospital, Taiyuan, Shanxi, China; ^2^Department of Pharmacology, Shanxi Medical University, Taiyuan, Shanxi, China

## Abstract

Adiponectin is a cytokine produced by adipocytes and acts as a potential cardioprotective agent and plays an important role in myocardial ischemia/reperfusion injury. In a myocardial hypoxia/reoxygenation model using neonatal rat ventricular myocytes, we investigated the contribution of adiponectin-mediated autophagy to its cardioprotective effects. Cardiomyocytes were exposed to hypoxia/reoxygenation pretreated with or without adiponectin in the presence of absence of rapamycin. Cell viability was analyzed using the 3-(4,5-dimethylthiazol-2-yl)-2,5-diphenyltetrazolium bromide method. Western blotting assay was used to determine the expression levels of microtubule-associated proteins 1A/1B light chain 3B (LC3B), adenosine monophosphate-activated protein kinase (AMPK), mammalian target of rapamycin (mTOR), p62/sequestosome 1, unc-51 like autophagy activating kinase 1 (ULK1), and Beclin-1. Autophagosome formation was detected by monodansylcadaverine staining. We found that hypoxia induced a time dependent decline in cardiomyocyte viability, and increase in autophagy and reoxygenation further augmented hypoxia-induced autophagy induction and consequently reduced cell viability. Adiponectin treatment alleviated hypoxia/reoxygenation-induced cellular damage and autophagy in cardiomyocytes. Adiponectin treatment also attenuated hypoxia/reoxygenation-promoted cardiomyocyte autophagy even in the presence of another autophagy stimulator rapamycin in part by inhibiting vacuolar hydron-adenosine triphosphatase. Additionally, autophagy suppression by adiponectin during hypoxia/reoxygenation was associated with the attenuated phosphorylation of AMPK and ULK1, augmented phosphorylation of mTOR, and the reduced protein expression levels of Beclin-1 in cardiomyocytes. Taken together, these results suggest that adiponectin protects ischemia/reperfusion-induced cardiomyocytes by suppressing autophagy in part through AMPK/mTOR/ULK1/Beclin-1 signaling pathway.

## 1. Introduction

Ischemic heart disease remains a significant health problem and continuously receives attentions globally [1]. Myocardial ischemia/reperfusion injury is largely unavoidable and seriously influences clinical therapeutic efficacy [2, 3]. Ischemia/reperfusion-induced cardiomyocyte injury involves several interrelated factors such as calcium overload and excessive reactive oxygen production leading to cell injury via necrosis, apoptosis, and accelerated autophagy [4, 5]. Among them, excessive autophagy is associated with ischemia/reperfusion-induced myocardial injury in the patients with increased oxidative stress [6]. Therefore, understanding the role and potential mechanisms in the pathogeneses of myocardial ischemia/reperfusion injury may help establish a novel strategy for pharmacologically treating ischemia/reperfusion-induced myocardial injury.

Autophagy is a biological process that maintains tissue homeostasis by degrading damaged organelles, misfolded proteins, and pathogens in membrane vesicles [4, 7]. Autophagy is remarkably enhanced under certain physiological conditions [8]. Though autophagy is augmented during ischemia/reperfusion-induced cardiomyocyte injury and protects cardiomyocytes from ischemia stress, persistent autophagy activation is also detrimental [6, 9]. Despite beneficial role of autophagy in cell survival under certain circumstances, its dual roles on cardiomyocytes are also substantially linked to the pathogenesis of ischemia/reperfusion-induced cardiomyocyte injury [6, 9]. Therefore, optimally inhibiting hyperactivated autophagy may help reduce ischemia/reperfusion-induced myocardial damage.

Adiponectin is a cytokine predominantly produced by adipocytes [10, 11] and present as a full-length 30 kDa protein or a C-terminal globular domain (known as globular adiponectin) with an equivalent biological activity [12, 13]. Adiponectin is involved in many physiological processes including the regulation of lipid and glucose metabolism [14, 15]. In addition to protection against atherogenesis, diabetogenesis, and apoptosis, adiponectin also protected cardiomyocytes from ischemia/reperfusion attack [16, 17]. We have previously found that adiponectin suppressed vascular calcification by attenuating endoplasmic reticulum stress and alleviated hypoxia/reoxygenation-induced injury in a neonatal myocardial hypoxia/reoxygenation *in vitro* model [18, 19]. However, it has not been completely investigated whether autophagy plays a role in adiponectin-mediated cardioprotection.

Using the neonate rat cardiomyocyte hypoxia/reoxygenation *in vitro* model, we explored whether the protection of adiponectin on hypoxia/reoxygenation-induced cardiomyocyte injury is mediated by suppressing autophagy formation. Our study provides the evidence for targeting modulation of autophagy for treating ischemia/reperfusion-induced cardiomyocyte injury.

## 2. Materials and Methods

### 2.1. Rat Cardiomyocyte Culture

Neonatal Sprague-Dawley rats at 1–3 days of age were obtained from the SBF Biotechnology Co. Ltd., Beijing, China. Cardiomyocytes were prepared by digesting hearts with trypsin and type II collagenase as described previously [20, 21]. Briefly, neonates were anesthetized by inhalation of 1.5% isoflurane and oxygen. The heart was exposed through the left-side thoracotomy, removed, and digested with trypsin (0.5%(*w*/*v*), Sigma-Aldrich, USA) and type II collagenase (200 U/ml, Sigma-Aldrich, USA) at 37°C for 30 minutes with rotation. The single cells were suspended in Dulbecco's Modified Eagle Medium (DMEM, Hyclone Laboratories, Logan, Utah, USA) supplemented with 10% fetal bovine serum (FBS, Hyclone Laboratories), plated at 6.6 × 10^4^ cells/cm^2^ in the dishes, and cultured in a humidified atmosphere with 5% CO_2_ at 37°C. The use and care of animals in this study were approved by Shanxi Medical University Animal Care and Use Committee, Taiyuan, Shanxi, China, and conducted in compliance with the university guideline.

### 2.2. Hypoxia/Reoxygenation Procedure Culture

Neonate rat cardiomyocytes in a confluence of 80-90% were plated in high-glucose DMEM media (4.5 mg/ml) supplemented with 1% FBS and incubated for 24 h. Thereafter, cells were transferred to low-glucose DMEM media (1 mg/ml) and cultured in a tri-gas incubator (HERA cell VIOS 160i, Thermo, USA) at hypoxia condition (5% CO_2_, 1% O_2_, and 94% N_2_) at 37°C for indicated time. At the end, the cells were cultured at 5% CO_2_ at 37°C for various time varied by reoxygenation time. As the controls, cells were cultured at normoxia condition (5% CO_2_ and 37°C) in an identical procedure.

### 2.3. Treatment of Cardiomyocytes with Adiponectin, Rapamycin, and Bafilomycin A1

Recombinant rat adiponectin, rapamycin, and bafilomycin A1 were obtained from the Sigma-Aldrich, USA. Adiponectin was reconstituted in phosphate-buffered saline (PBS), and rapamycin and bafilomycin A1 were prepared freshly in dimethyl sulfoxide (DMSO). Cardiomyocytes were cultured in DMEM media and pretreated with 0.5, 1, and 2 *μ*g/ml adiponectin beginning 24 hours, or with either rapamycin (5 nM) or bafilomycin A1 (100 nM) one hour prior to hypoxia/reoxygenation. As the vehicle controls, cells were treated with equal value of PBS or DMSO in an identical regimen.

### 2.4. Measurement of Cardiomyocyte Viability

Mitochondrial succinodehydrogenase activity was used to assess cell viability via 3-(4,5-dimethylthiazol-2-yl)-2,5-diphenyltetrazolium bromide) (MTT) assay. Briefly, five thousand cardiomyocytes (1 ml) were seeded at in 96-well plates and incubated with MTT (5 mg/ml, Sigma-Aldrich) for 4 hours. Thereafter, culture medium was removed by centrifuge, and cells were lysed to dissolve formazan by adding 150 *μ*L DMSO. Cell viability was quantified by measuring absorbance (optical density) at 490 nm on a microplate reader and expressed as the fold change relative to that in vehicle control treatment (PBS) as described previously [20, 22].

### 2.5. Assessment of Cardiomyocyte Injury

Lactate dehydrogenase (LDH) release assay was employed to measure cardiomyocyte injury following hypoxia or hypoxia/reperfusion in the presence or absence of indicated treatments using a LDH assay kit (Solarbio Science & Technology Co. Ltd., Beijing, China) according to the manufacturer's instructions. Briefly, cardiomyocytes were cultured at a density of 5 × 10^6^ cells/ml and treated with indicated reagents. Culture supernatant was subjected to LDH assay by measuring absorbance at 490 nm using a spectrophotometer (Thermo Fisher Scientific, Fremont, CA, USA).

### 2.6. Detection of Autophagic Vacuole

Autophagic vacuole was determined by monodansylcadaverine (MDC) staining using a commercial kit (KGATG001, Nanjing KeyGen Biotech. Co., Ltd. Nanjing, Jiangsu, China) as described previously [23]. Cardiomyocytes were cultured in 6-well plates, reached 50-60% confluence (50-60%) and washed with washing buffer, and immersed in 400 *μ*L working solution for 20 min at room temperature in the dark. After three times washes with washing buffer, MDC staining was visualized and imaged on an Olympus fluorescence microscope (Olympus, Central Valley, PA, USA) at an excitation wavelength of 355 nm and an emission of 512 nm using ImageJ software (Version 1.8.0_172, National Institutes of Health, Bethesda, MD, USA). Autophagic vacuole was quantitated by measuring fluorescence staining intensity.

### 2.7. Western Blotting

Cardiomyocytes were lysed using cell lysis buffer. Equal amounts of proteins were used for western blotting as described previously [23, 24]. Briefly, proteins were denatured by boiling, separated by 12% sodium dodecyl sulfate-polyacrylamide gels, and transferred to polyvinylidene difluoride membranes (EMD Millipore, Burlington, MA, USA). Following blocking nonspecific binding with PBS containing 8% nonfat milk and 0.5% Tween-20 for 2 h, the membranes were sequentially incubated with a rabbit antibody against microtubule-associated proteins 1A/1B light chain 3B (LC3B), p62/sequestosome 1, adenosine monophosphate-activated protein kinase (AMPK), mammalian target of rapamycin (mTOR), unc-51 like autophagy activating kinase 1 (ULK1), Beclin-1, phosphorylated-AMPK, phosphorylated-mTOR, phosphorylated-ULK1, or glyceraldehyde-3-phosphate dehydrogenase (GAPDH) (all antibodies used at 1 : 1000 dilution) and washed and incubated with goat anti-rabbit secondary antibody-horseradish peroxidase conjugate (1 : 3000 dilution). Proteins of interest were visualized using the enhanced chemiluminescence kit, and each band density was quantified by a densitometry. Except for polyvinylidene difluoride membranes and enhanced chemiluminescence kit (EMD Millipore), all reagents and materials were obtained from the Cell Signaling Technology, Danvers, MT, USA.

### 2.8. Statistical Analysis

GraphPad Prism 6.0 (GraphPad Software, San Diego, CA, USA) was used for data analysis. All data are given as mean and standard error of mean (SEM). Unpaired Student's *t*-test and one or two-way analysis of variance (ANOVA) followed by Bonferroni correction were utilized for the two group comparison, respectively. The *P* value less than 0.05 was considered statistically significant.

## 3. Results

### 3.1. Hypoxia and Reoxygenation Is Synergized in Inducing Autophagy in Cardiomyocytes

**Figure 1:**
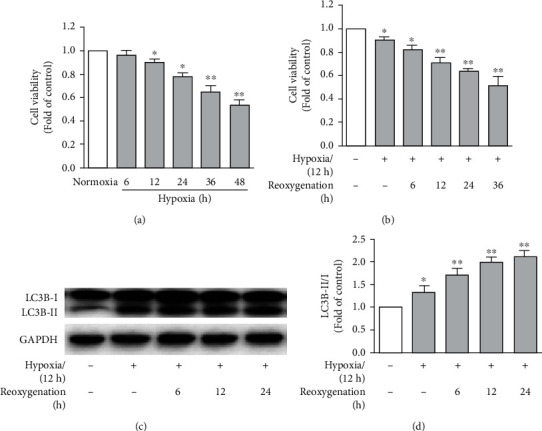
Hypoxia/reoxygenation reduces cell viability and increases autophagy in neonate rat cardiomyocytes. Neonate rat cardiomyocytes were cultured in normoxia (control), hypoxia condition for 6-48 hours alone, or followed by reoxygenation for 6-24 hours. Cell viability was measured by MTT assay (a, b; *n* = 6), or cell lysates were subjected to Western blotting analysis to detect autophagy (c, d; *n* = 4). All data on cell viability or autophagy formation in hypoxia or hypoxia/reoxygenation are calculated as the fold changes relative to normoxia culture (control) and presented as mean and standard error of mean. (a) Cell viability measured following normoxia or hypoxia culture. (b) Cell viability measured following normoxia culture, hypoxia alone (12 hours), or hypoxia followed by reoxygenation for 6-36 hours. (c) Representative Western blotting images for LC3B-I, LC3B-II, and GAPDH in cardiomyocytes cultured at normoxia condition, hypoxia alone for 12 hours, or followed by reoxygenation for 6-24 hours. (d) Quantification of autophagy formation in cardiomyocytes in indicated culture conditions by the ratio of LC3B-I to LC3B-II. ^∗^*P* < 0.05 and ^∗∗^*P* < 0.01, one-way (a) or two-way (c, d) ANOVA followed by comparison with normoxia culture condition (control).

As the first step, we used MTT assay and evaluated neonate cardiomyocyte viability following hypoxia exposure (Figure 1(a)). Hypoxia induced a time dependent decline in cardiomyocyte viability, with a significant reduction beginning 12 hours after hypoxia exposure. Then, we fixed hypoxia exposure time as 12 hours and assessed the influence of various length of reoxygenation on cardiomyocyte viability (Figure 1(b)). Reoxygenation resulted in further reduction in hypoxia-induced cell death with increasing reoxygenation time. A significant reduction in cardiomyocyte viability was noted even after 6-hour reoxygenation as compared to 12-hour hypoxia exposure alone. Hypoxia followed by reoxygenation caused an approximately 30% and 50% cell death after 12 and 48 hours, respectively, as compared to control cells cultured at normoxia condition.

Next, we determined whether hypoxia alone or hypoxia/reoxygenation induces autophagy in cardiomyocytes. LC3B consists of LC3B-I and LC3B-II and regulates autophagy [23, 25]. Upon autophagy activation, cytosolic form LC3B-I is converted to membrane-bound form LC3B-II leading to increased ration of LC3B-II to LC3B-I, which served as marker of autophagy [23, 25]. In normoxia condition, western blot analysis detected weak signaling for autophagy activation as indicated by the presence of membrane-bound form LC3B-II. Following 12-hour hypoxia exposure, autophagy activation was remarkably augmented in cardiomyocytes as evidenced by increased LC3B-II (Figure 1(c)) as well as the ratio of LC3B-II to LC3B-I (Figure 1(d)). Subsequent reoxygenation after hypoxia exposure promoted further autophagy activation in cardiomyocytes in a time-dependent manner, with a 2-fold increase in the ratio of LC3B-II to LC3B-I proteins in cardiomyocytes beginning 12-hour reoxygenation as compared to normoxia culture condition (Figures 1(c) and 1(d)). Thus, reoxygenation augmented hypoxia-induced autophagy induction and consequently reduced cell viability. Based on these results, all experiments related to hypoxia/reoxygenation in this study were conducted by 12-hour hypoxia followed by 12-hour reoxygenation unless indicated.

### 3.2. Adiponectin Treatment Attenuates Hypoxia/Reoxygenation-Induced Autophagy in Cardiomyocytes

**Figure 2:**
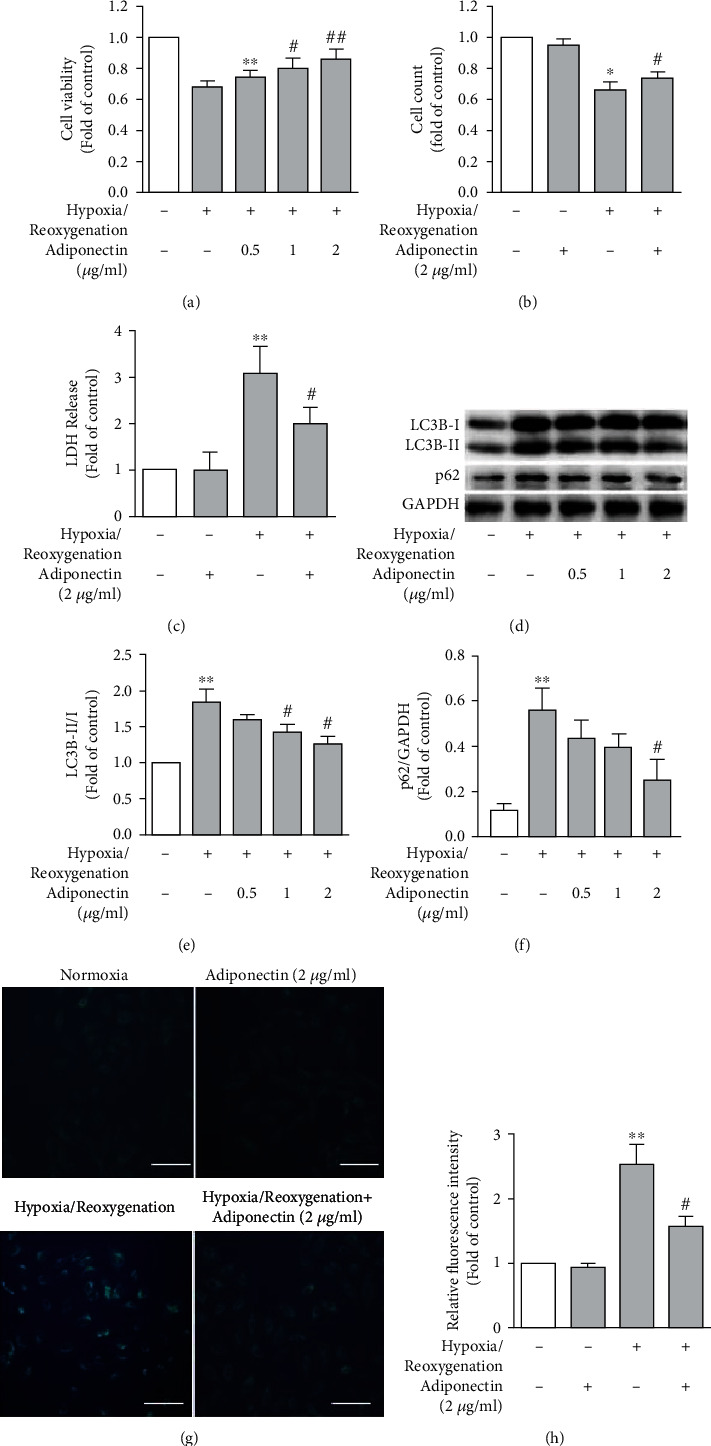
Adiponectin treatment attenuates cardiomyocyte injury and autophagy following hypoxia/reoxygenation. Neonate rat cardiomyocytes were treated cultured at normoxia condition, hypoxia for 12 hours alone, or followed by reoxygenation for 12 hours in the presence of adiponectin (0.5-2.0 mg/ml) or vehicle PBS (phosphate-buffered saline). Cell viability or cell injury was measured by MTT or cell counting, or cell injury (all; *n* = 6). Autophagy was detected by Western blotting analysis or monodansylcadaverine (MDC) staining (g, h; *n* = 4). All data on cell viability (a, b), cell injury (c), or autophagy formation (e, f, and h) in hypoxia or hypoxia/reoxygenation are calculated as the fold changes relative to normoxia culture (control) and presented as mean and standard error of mean. (a, b) Cell viability measured at indicated conditions measured via MTT assay (a) or cell counting (b). Cell viability in hypoxia or hypoxia/reoxygenation is calculated as the fold changes relative to normoxia culture (control). (c) Cell injury at indicated culture conditions measured via LDH release assay. Cell injury in hypoxia or hypoxia/reoxygenation is calculated as the fold changes relative to normoxia culture (control). (d) Representative Western blotting images for the expression of autophagy-related proteins (LC3B and p62) and GAPDH in cells at different conditions with or without adiponectin treatment. (e) Quantification of autophagy via the ratio of LC3B-I to LC3B-II as the fold changes in relative to vehicle-treated cells at normoxia culture condition. (f) Quantification of autophagy protein p62 as the ratio of p62 to GAPDH protein in cells at indicated conditions. (g) Representative immunofluorescence staining images for autophagic vacuoles detected via monodansylcadaverine staining. Scale bar, 100 *μ*m. (h) Quantification of autophagy formation via autophagic vacuoles. Data are fluorescence intensity in differentially treated cells in relative to vehicle-treated cells at normoxia culture condition. In (a, b, c, e, f, and h), all data are mean and standard error of mean. Two-way ANOVA followed by two group comparison was used for statistically significant testing. ^∗^*P* < 0.05 and ^∗∗^*P* < 0.01 compared to normoxia culture with vehicle treatment (control); ^#^*P* < 0.05 and ^##^*P* < 0.01 compared to PBS-treated cells followed by 12-hour hypoxia/12-hour reoxygenation.

Next, we aimed to study the role of autophagy in adiponectin-mediated protection in hypoxia/reoxygenation-induced cardiomyocyte injury. As revealed by the MTT, cell counting, and LDH release assays (Figures 2(a)–2(c)), pretreatment with adiponectin at various doses remarkably increased cardiomyocyte cell viability and reduced cardiomyocyte injury following hypoxia/reoxygenation, with maximal effect noted for adiponectin at 2 mg/ml. Under normoxia condition, adiponectin treatment alone had no recognizable effect on either cardiomyocyte viability or LDH release. These results verified our previous studies that adiponectin attenuates hypoxia/reoxygenation-induced cardiomyocyte injury.

In the Western blotting assay, hypoxia/reoxygenation dramatically induced autophagy in cardiomyocytes as indicated by increased LC3B-II as well as the ratio of LC3B-II to LC3B-I proteins as compared to normoxia controls (Figures 2(d) and 2(e)). In contrast, pretreatment with adiponectin dose-dependently ameliorated autophagy in cardiomyocytes, with maximal inhibition noted for 2 *μ*g/ml adiponectin. p62/sequestosome 1, an autophagy-associated protein, was also substantially increased in cardiomyocytes following hypoxia/reoxygenation (Figure 2(f)). Pretreatment of cardiomyocytes with adiponectin was capable of reducing the expression levels of p62 protein as compared to vehicle-treated cardiomyocytes exposed to hypoxia/reoxygenation (Figure 2(f)). Further, we assessed the influence of adiponectin on autophagosome formation by the measurement of acidophilic autofluorescent compounds in autophagic vacuoles via MDC staining [23]. More autophagic vacuoles were found in cardiomyocytes following hypoxia/reoxygenation as compared to cardiomyocytes under normoxia control culture. However, treatment with adiponectin markedly reduced the number of autophagic vacuoles induced by hypoxia/reoxygenation as compared to vehicle treatment (Figure 2(g)). Further semiquantification of autophagosome-derived fluorescence indicated a more than 50% reduction in phagosome formation in adiponectin-treated cardiomyocytes following hypoxia/reoxygenation as compared to vehicle treatment (Figure 2(h)). Collectively, our results from three distinct and complementary assays demonstrate the induction of autophagy by hypoxia/reoxygenation and its suppression by adiponectin in cardiomyocytes.

### 3.3. Adiponectin Treatment Suppresses Rapamycin-Augmented Autophagy in Cardiomyocytes following Hypoxia/Reoxygenation

**Figure 3:**
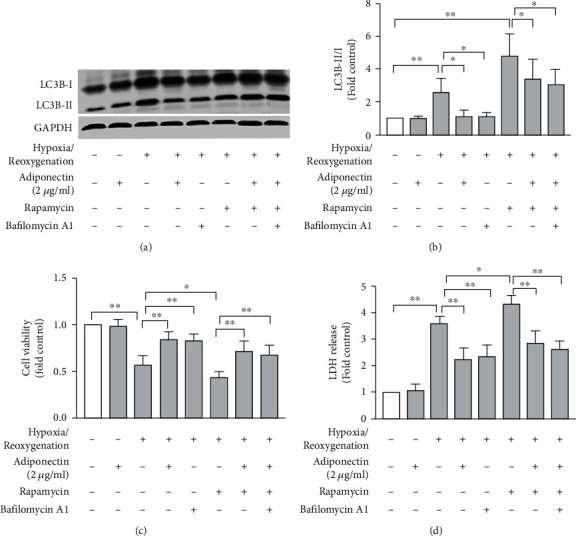
Adiponectin treatment attenuates rapamycin and hypoxia/reoxygenation-augmented autophagy in cardiomyocytes and has no additive effect with vacuolar H^+^-ATPase inhibitor bafilomycin. Neonate rat cardiomyocytes were cultured at normoxia or hypoxia condition (12 hours) or underwent 12 hour-hypoxia/12 hours-reoxygenation in the presence of vehicle control, adiponectin (2 mg/ml), rapamycin (5 nM), or bafilomycin (100 nM). Cell viability, cell injury, and the protein levels of LC3B-I and LC3B-II were determined by MMT, LDH release, and Western blotting analyses, respectively. (a) Representative Western blotting images for the proteins of LC3B-I, LC3B-II, and GAPDH in cells at different experiment settings. (b) Quantification of autophagy as the ratios of LC3B-II/LC3B-I in cardiomyocytes receiving differential treatments (*n* = 4/group). (c, d) Mean and standard error of mean of cardiomyocyte viability and injury measured via MTT and LDH release assays, respectively (*n* = 6/group). Two-way ANOVA followed by two group comparison. ^∗^*P* < 0.05 and ^∗∗^*P* < 0.01 between two groups.

Rapamycin is a well-known autophagy inducer [26]. We investigated whether rapamycin modulates autophagy in cardiomyocytes following hypoxia/reoxygenation. As evidenced by the increased ratio of LC3BII to LC3B-I (Figures 3(a) and 3(b)), rapamycin treatment resulted in further augmentation of hypoxia/reoxygenation-induced autophagy formation in cardiomyocytes as compared to vehicle treatment. However, treatment with adiponectin caused an approximately 50% reduction in rapamycin-promoted autophagy in cardiomyocytes under hypoxia/reoxygenation condition (Figures 3(a) and 3(b)). Consistent with the influence on autophagy formation, rapamycin treatment further reduced hypoxia/reoxygenation-induced cardiomyocyte viability and injury as evaluated by MMT and LDH release assays, which were counteracted by adiponectin (Figures 3(c) and 3(d)). In contrast, bafilomycin A1, which specifically and reversibly inhibits autophagy formation by inhibiting vacuolar hydron-adenosine triphosphatase (H^+^-ATPase), had no additional suppression on autophagy formation in adiponectin-treated cardiomyocytes following hypoxia/reoxygenation regardless of whether rapamycin was present or absent (Figures 3(a) and 3(b)). Similarly, bafilomycin A1 treatment had no additive effect with adiponectin in increasing cell viability or suppressing cell injury as indicated by MTT and LDH release tests, respectively (Figures 3(c) and 3(d)). These results suggest that adiponectin attenuated hypoxia/reoxygenation-induced autophagy even in the presence of an additional autophagy stimulator such as rapamycin in part by inhibiting vacuolar H^+^-ATPase.

### 3.4. Adiponectin Treatment Modulates AMPK and Its Downstream Signaling in Cardiomyocytes following Hypoxia/Reoxygenation

AMPK induces autophagy by phosphorylating mTOR and ULK1 [23, 25, 27]. Beclin-1, as downstream molecule of ULK1, contributes to autophagy formation in many cells including cardiomyocytes [23, 28, 29]. Thus, we examined whether hypoxia/reoxygenation alters the phosphorylation or expression levels of AMPK, mTOR, ULK1, and Beclin-1 in cardiomyocytes in the presence of adiponectin. In the Western blotting analysis, the phosphorylated levels of AMPK and ULK1 were elevated, whereas the phosphorylated levels of mTOR were diminished, in cardiomyocytes following hypoxia/reoxygenation as compared to cardiomyocytes cultured in normoxia condition. Beclin-1 expression levels were substantially increased in cardiomyocytes following hypoxia/reoxygenation as compared to cardiomyocytes cultured under normoxia condition.

In normoxia condition, pretreatment with adiponectin did not alter the phosphorylation of AMPK, mTOR, and ULK1 as well as Beclin-1 protein expression levels. In contrast, pretreatment with adiponectin abrogated hypoxia/reoxygenation-induced phosphorylation of AMPK and ULK1 in cardiomyocytes. Adiponectin treatment completely negated the suppression of mTOR phosphorylation in cardiomyocytes following hypoxia/reoxygenation. Additionally, upregulated expression of Beclin-1 protein by hypoxia/reoxygenation was almost completely abolished by adiponectin treatment. Altogether, these results suggest that the suppression of hypoxia/reoxygenation-induced autophagy by adiponectin was in part mediated by AMPK/mTOR/ULK1/Beclin-1 signaling pathway.

## 4. Discussion

In this study, we reconfirmed the beneficial effect of adiponectin in cardiomyocytes following hypoxia/reoxygenation. Reoxygenation was synergized with hypoxia in inducing autophagy formation in a reoxygenation time-dependent manner as indicated by increased ratio of LC3B II to LC3B I proteins, upregulated p62 protein expression levels, and increased autophagic vacuoles. Adiponectin was effective in suppressing autophagy formation by either hypoxia/reoxygenation exposure or treatment with mTOR inhibitor rapamycin. Cotreatment with vacuolar H^+^-ATPase inhibitor bafilomycin A1, an autophagy inhibitor, did not result in the additional suppression of adiponectin-treated cardiomyocytes following hypoxia/reoxygenation or rapamycin treatment.

Ischemia/reperfusion injury is the major cause of ischemic heart diseases [4, 30], mitigating cardiomyocyte damage and consequently preserving heart function are thus critical for reducing the risk for cardiovascular diseases [4].

Cardiomyocyte autophagy following ischemia/reperfusion could be adaptive or maladaptive thus being protective or pathogenic in heart diseases [31, 32]. In our study, autophagy formation increased with decreased cell viability and reducing cell injury (Figure 1). Further, adiponectin dose-dependently diminished cardiomyocyte autophagy formation following hypoxia/reoxygenation in conjunction with increased cell viability and reducing cell injury as assessed by MTT and LDH release tests (Figure 2). Adiponectin also suppressed rapamycin-promoted cardiomyocyte autophagy formation and cell injury and increased cell viability after hypoxia/reoxygenation (Figure 3). Taken together, all our data suggest that autophagy induction after hypoxia/reoxygenation exposure was detrimental to rather than protective against cardiomyocytes. In support our assumption, it has been suggested that ischemia/reperfusion impaired injury autophagic flux leading to ineffectiveness in clearing autophagosome [33]. Consequently, excess autophagosome accumulation may result in mitochondrial calcium overload, excessive generation of reactive oxygen speciose, and eventually cell death [33, 34].

**Figure 4:**
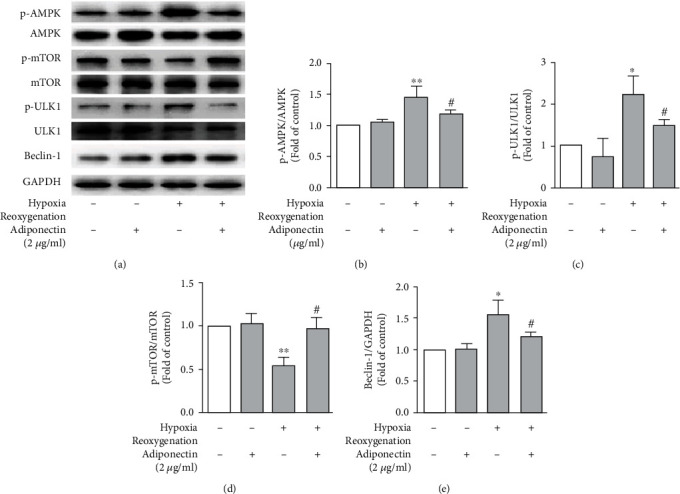
Adiponectin treatment counteracts hypoxia/reoxygenation-induced AMPK activation and downstream signaling in cardiomyocytes. Neonate rat cardiomyocytes were cultured at normoxia condition (control group) or underwent hypoxia/reoxygenation exposure in the presence of adiponectin (2 *μ*g/ml) or vehicle. (a) Representative western blot images showing the total and phosphorylated form of AMPK, mTOR, and ULK11 as well as Beclin-1 protein. (b–e) Quantification of phosphorylated AMPK (b), ULK1 (c), and mTOR (d) as well as Beclin-1 protein levels. Phosphorylated levels of individual proteins were presented as the fold changes relative to that in vehicle-treated cells cultured at normoxia condition (control group). All data are mean and standard error mean (*n* = 4). Two-way ANOVA followed by two group comparison. ^∗^*P* < 0.05 and ^∗∗^*P* < 0.01 compared to control group; ^#^*P* < 0.05 compared to hypoxia/reoxygenation group.

AMPK regulates autophagy formation by preventing and increasing mTOR and ULK1 phosphorylation, respectively [35–37]. It has been previously shown that the absence of nutrients and energy stimulated autophagy in association with suppression of mTOR signaling [23, 38]. ULK1 regulates early stages of autophagy in response to starvation or mTOR inhibition [27]. In contrast to AMPK, phosphorylated mTOR was inhibitory to ULK1. Addition, Beclin-1, a downstream molecule of ULK1, also contributed to autophagosome formation in cardiac myocytes [23, 39]. In our study, hypoxia/reoxygenation increased the phosphorylation of AMPK and ULK1 whereas suppressed the phosphorylation of mTOR in cardiomyocytes (Figure 4). Consequently, the expression levels of Beclin-1 protein, downstream of ULK1 in autophagic pathway levels, were elevated in cardiomyocytes following hypoxia/reoxygenation (Figure 4). All these effects were however almost completely reversed following adiponectin treatment. Collectively, all our data suggest that adiponectin protected cardiomyocytes against hypoxia/reoxygenation injury in part by attenuating the AMPK/mTOR/UKL1/Beclin-1 autophagic pathways.

Additionally, vacuolar H^+^-ATPase by its proton pumping activity activates lysosomal acid hydrolases, which are responsible for degrading damaged organelles, misfolded proteins, and pathogens in lysosomes delivered from autophagosomes [40]. In the present study, inhibiting vacuolar H^+^-ATPase by bafilomycin A1 did not lead to further autophagy inhibition in adiponectin-treated cardiomyocyte following hypoxia/reoxygenation regardless of whether rapamycin was added. These results suggest that adiponectin and bafilomycin A1 may suppress hypoxia/reoxygenation-augmented autophagy in cardiomyocytes via a common mechanism probably targeting vacuolar H^+^-ATPase. Thus, in addition to the AMPK/mTOR/UKL1/Beclin-1 pathway, vacuolar H^+^-ATPase may be alternative target by which adiponectin suppressed autophagy.

In this study, we focused on the direct effect of adiponectin-mediated autophagy inhibition in hypoxia/reoxygenation-induced cardiomyocyte injury *in vitro*. However, immune response particularly innate immunity mediated by macrophages and neutrophils is critical for the initiation and resolution of ischemia/reperfusion triggered cardiac injury [41, 42]. Thus, adiponectin may also protect cardiac injury by modulating immune cell recruitment and activity *in vivo*. For example, studies employing genetic deficiency of or pharmacological intervention with adiponectin have shown that adiponectin negatively regulated the endothelial expression of E-selectin, vascular cell adhesion molecule-1, and intracellular adhesion molecule-1 all essential for leukocytes into inflamed tissues [43–47]. In high fat diet-fed LDL receptor-deficient mice, adiponectin overexpression by adenoviral gene transfer attenuated macrophage burden and the levels of proinflammatory mediators such as tumor necrosis factor-*α*, interleukin-6, interleukin-12, and C-C motif chemokine ligand 2 whereas increased anti-inflammatory interleukin-10 in the aortas after subcutaneous angiotensin II infusion [48]. Additionally, adiponectin treatment promoted anti-inflammatory alternative macrophage activation but attenuated proinflammatory neutrophil extracellular trap formation [49–52].

In conclusion, the present study found that adiponectin protected cardiomyocytes from hypoxia/reoxygenation injury in part by inhibiting AMPK/mTOR/ULK-1/Beclin-1 and probably vacuolar H^+^-ATPase autophagy pathways. Our study reinforces the notion that adiponectin may serve as an effective therapeutic strategy for mitigating ischemia/reperfusion-induced myocardial injury in patients with cardiovascular diseases.

## Data Availability

The data used to support the findings of this study are included within the article.
